# Supplementary material for: Effect of matcha green tea on cognitive functions and sleep quality in older adults with cognitive decline: A randomized controlled study over 12 months
Source: PLoS One. 2024 Aug 30;19(8):e0309287. doi: 10.1371/journal.pone.0309287 (PMC11364242; doi:10.1371/journal.pone.0309287)
Supplement: S1 File — (PDF) [file pone.0309287.s001.pdf]

Supporting Materials & Methods

Materials & Methods

1. Study design

1.1 Recruiting of participants

Participants were selected according to the following criteria: (1) Japanese-speaking men and women aged 60–85 years living with a study partner responsible for checking capsule intake and escorting visitors to the hospital, (2) presence of mild cognitive impairment (MCI) or subjective cognitive decline (SCD) in the University of Tsukuba Hospital (Tsukuba, Ibaraki, Japan) and Memory Clinic (Toride, Ibaraki, Japan).

1.2. Demographics and medical issues

Information on age, sex, education, occupation, marital status, family members, daily activities, previous medical and psychiatric diseases, medication, alcohol, and smoking as dementia risk factors were included in the questionnaire.

1.3. Assessment of mood status

To assess depression, a 15-item short version of the Geriatric Depression Scale for mood evaluation was required [1]. The participants who scored six were considered to have depressive symptoms.

1.4. Complaints of memory loss

Nineteen items from the Deterioration de Cognitive Observed were conducted to decide whether participants had memory difficulties. If the participants indicated problems on ≥1 item, memory difficulties were present.

1.5. Assessment of cognition functions

All the participants underwent an assessment named the 5-Cog, which used a set of

tests to measure five cognitive domains: attention, memory, visuospatial function, language, and reasoning [2]. The 5-Cog cognitive assessment was conducted by an examiner for a group of 50 participants (maximum) and with the use of a projector. All the participants were asked to record their answers on an answer sheet. Mean duration of the 5-Cog examination was 35 min. For participants who had difficulty understanding the tasks or impaired hearing or vision, the 5-Cog examination was individualized in a face-to-face setting. During the interview, the participants who could not respond to our instructions or to some of the scales because of obvious cognitive impairment were also identified.

2. Sample size

2-1. Statistical evaluation for the number of cases

The required sample size was calculated using G*Power3 software (Heinrich Heine University, Düsseldorf, Germany). Details are described below [3]. It was decided to test the difference between the medians of the two groups without correspondence by the Wilcoxon-Mann-Whitney test (two groups), and the result of setting "two-tailed, logistic distribution, effect size = 0.7, significance level = 0.05, power = 0.8" as shown in Figure below, it was determined that 31 cases were required for each group. Therefore, the target sample size of cases was set as follows. If 7 of the 40 cases in each group would drop out, the target was set at 33 cases in each group, and the validity of this target was examined.

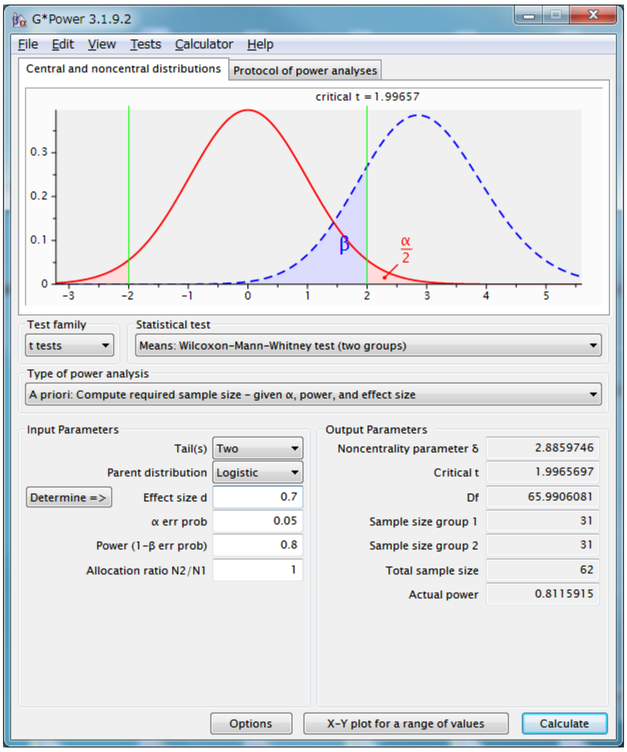

(1) Setting up a statistical model

Baseline and post-intervention values for the intervention and control groups were denoted as follows:

$$X_{\mathrm{g}} = \text{baseline value of group g},\ Y_{\mathrm{g}} = \text{Post} - \text{intervention value of group g}$$

The pre- and post-intervention values were assumed to follow the following bivariate normal distribution.

$$\begin{pmatrix} X_g \\ Y_g \end{pmatrix} \sim N\left(\begin{pmatrix} \mu_{gX} \\ \mu_{gY} \end{pmatrix}, \begin{bmatrix} \sigma_X^2 & \rho\sigma_X\sigma_Y \\ \rho\sigma_X\sigma_Y & \sigma_Y^2 \end{bmatrix} \right)$$

The measure of effectiveness evaluation was defined as the amount of change between pre- and post-intervention, denoted as $\Delta_{\mathrm{g}} = X_{\mathrm{g}} - Y_{\mathrm{g}}$ for group g.

The mean and variance of the change Δ_{g} were given by:

$$E(\Delta_g) = \mu_{gX} - \mu_{gY}$$
$$V(\Delta_{\mathrm{g}}) = \sigma_X^2 - 2\rho\sigma_X\sigma_Y + \sigma_Y^2$$

(2) Setting of detection power calculation

Assume equal variances for the pre- and post-intervention, $\sigma_X^2 = \sigma_Y^2 = \sigma^2$. Also, the change in the control group be $\Delta_0 = X_0 - Y_0$, and assume $E(\Delta_0) = \mu_{0X} - \mu_{0Y} = 0$. Similarly, let $E(\Delta_1)$ be mean change in the intervention group, and assume that the baseline mean values for both groups are assumed to be equal $(\mu_{0X} = \mu_{1X} = \mu_X)$. Since the number of cases in each group was fixed, the expected effect and unknown parameters (μ_X, σ^2, ρ) and the power of the T-test for comparing the change between groups was determined.

(μ_Y, σ^2) was estimated by integrating the results of multiple papers retrieved beforehand. Correlations between pre- and post-intervention effect measures ρ and the average change in the intervention group $E(\Delta_1) = \mu_{1X} - \mu_{1Y}$ values were given and power was calculated for each value.

(3) Calculation of baseline mean and variance (meta-analysis)

$T_i, v_i$is the paper$i$ as the baseline mean and variance of the integrated meanμ_X was,

$$\mu_X = \frac{\sum_i w_i T_i}{\sum_i w_i}$$

However, it was determined through a weighted average using the reciprocals of variances represented by the formula:

$$w_i = \frac{1}{v_i}$$

The $\left(1 - {}^{\alpha}/_{2}\right)$ The % confidence interval for μ_X was $\mu_X \pm z\left(1 - {}^{\alpha}/_{2}\right) se(\mu_X)$

wherese $(\mu_X) = \sigma = \sqrt{{}^{1}/_{\sum w_i}}$

Results of papers to be integrated. The results of the meta-analysis yielded an estimate of $(\mu_X, \sigma) = (19.63, 0.86)$.

Reference No.	number of cases	group	baseline MoCA average	SD
[4]	142	intervention	22.98	3.36
[4]	72	contrast	22.2	3.54
[5]	41	intervention	22.5	4.5
[6]	30	intervention	21.37	2.04
[6]	30	contrast	18.43	1.06

(4) Calculation of detection power

Hypothesis was, $H: \Delta_1 - \Delta_0 = 0\ vs. K: \Delta_1 - \Delta_0 = \delta$

The test statistic was,

$$T = \frac{\delta}{\sqrt{V(\Delta_1 - \Delta_0)}}$$

Whereas,

$$V(\Delta_1 - \Delta_0) = 2V(\Delta_1)$$

$$= 4\sigma^2(1-\rho)\ \left[from\ V(\Delta_g) = \sigma_X^2 - 2\rho\sigma_X\sigma_Y + \sigma_Y^2, \sigma_X^2 = \sigma_Y^2 = \sigma^2\right]$$

rho	*(1-ρ)*	$4\sigma^2(1-\rho)$
-0.8	1.8	5.3251
-0.7	1.7	5.0293
-0.6	1.6	4.7334
-0.5	1.5	4.4376
-0.4	1.4	4.1418
-0.3	1.3	3.8459
-0.2	1.2	3.5501
-0.1	1.1	3.2542
0	1.0	2.9584
0.1	0.9	2.6626
0.2	0.8	2.3667
0.3	0.7	2.0709
0.4	0.6	1.7750
0.5	0.5	1.4792
0.6	0.4	1.1834
0.7	0.3	0.8875
0.8	0.2	0.5917

The Figure below displays power on the y-axis plotted against the standard deviation (σ) of the test statistic on the x-axis. Additionally, the expected effect (μ1) is represented on the z-axis in a three-dimensional plot. An SD of 3.5 for the effect measure of 3.5 indicated a group difference of 2.5 or greater in the amount of change, while an SD of 4.0 or greater corresponds to a group difference of 3.0 or more. This latter difference was significant when there were at least 33 cases in each group. Notably, a group difference of approximately 3.0 was observed in the reference literature results.

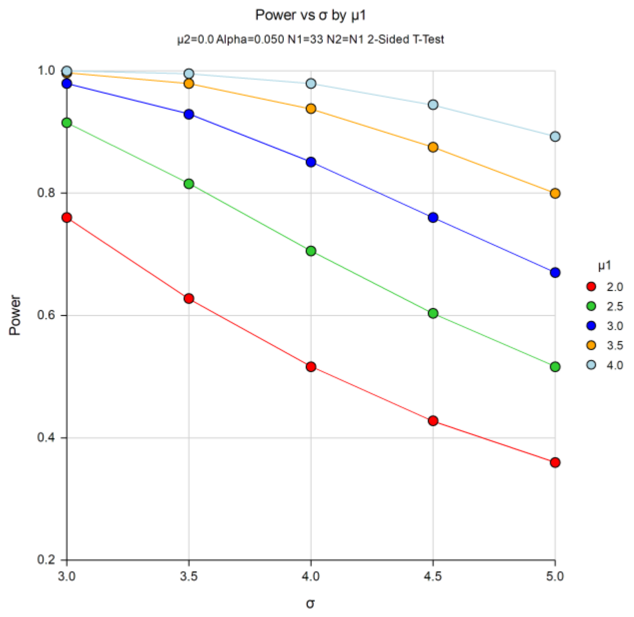

The graph presents a bivariate analysis with the horizontal axis representing variability, quantified as standard deviation (SD), and the vertical axis denoting statistical power. Diverse effects are distinguished by a unique color coding, each corresponding to the magnitude of a group difference in change over time. An inverse relationship is depicted between effect size and variability; as the effect size diminishes and/or variability increases, there is a concomitant decrease in the power to detect true effects statistically.

(5) Consideration

Although the efficacy indices in this study vary slightly from those in previous research [4–6], observing the anticipated effects outlined in the meta-analysis would render the data from all 66 patients a significant evidence base for preparing the subsequent validation study.

Numeric Results for Two-Sample T-Test Assuming Equal Variance

Alternative Hypothesis: H1: $\delta = \mu 1 - \mu 2 \neq 0$

Power	N1	N2	N	μ1*	μ2	δ	σ	Alpha
0.76028	33	33	66	2.0	0.0	2.0	3.0	0.050
0.62790	33	33	66	2.0	0.0	2.0	3.5	0.050
0.51620	33	33	66	2.0	0.0	2.0	4.0	0.050
0.42799	33	33	66	2.0	0.0	2.0	4.5	0.050
0.35979	33	33	66	2.0	0.0	2.0	5.0	0.050
0.91525	33	33	66	2.5	0.0	2.5	3.0	0.050
0.81532	33	33	66	2.5	0.0	2.5	3.5	0.050
0.70559	33	33	66	2.5	0.0	2.5	4.0	0.050
0.60365	33	33	66	2.5	0.0	2.5	4.5	0.050
0.51620	33	33	66	2.5	0.0	2.5	5.0	0.050
0.97934	33	33	66	3.0	0.0	3.0	3.0	0.050
0.92909	33	33	66	3.0	0.0	3.0	3.5	0.050
0.85095	33	33	66	3.0	0.0	3.0	4.0	0.050
0.76028	33	33	66	3.0	0.0	3.0	4.5	0.050
0.67024	33	33	66	3.0	0.0	3.0	5.0	0.050
0.99660	33	33	66	3.5	0.0	3.5	3.0	0.050
0.97934	33	33	66	3.5	0.0	3.5	3.5	0.050
0.93828	33	33	66	3.5	0.0	3.5	4.0	0.050
0.87526	33	33	66	3.5	0.0	3.5	4.5	0.050
0.79970	33	33	66	3.5	0.0	3.5	5.0	0.050
0.99963	33	33	66	4.0	0.0	4.0	3.0	0.050
0.99549	33	33	66	4.0	0.0	4.0	3.5	0.050
0.97934	33	33	66	4.0	0.0	4.0	4.0	0.050
0.94476	33	33	66	4.0	0.0	4.0	4.5	0.050
0.89261	33	33	66	4.0	0.0	4.0	5.0	0.050

*μ1 denotes the anticipated change in the intervention group, whereas σ refers to the standard deviation of the differences in change between groups.

References

1. Guerin JM, Copersino ML, Schretlen DJ. Clinical utility of the 15-item geriatric depression scale (GDS-15) for use with young and middle-aged adults. J Affect Disord. 2018;241: 59–62.

2. Yatomi N, Asada T. A mass cognitive test for the elderly: Development of Five Cog Test. Psychogeriatrics. 2006;17: 174.

3. Chow S-C, Wang H, Shao J. Sample Size Calculations in Clinical Research, Second Edition. CRC Press; 2007.

4. Tsolaki M, Kounti F, Agogiatou C, Poptsi E, Bakoglidou E, Zafeiropoulou M, et al. Effectiveness of nonpharmacological approaches in patients with mild cognitive impairment. Neurodegener Dis. 2011;8: 138–145.

5. Marzolini S, Oh P, McIlroy W, Brooks D. The effects of an aerobic and resistance exercise training program on cognition following stroke. Neurorehabil Neural Repair. 2013;27: 392–402.

6. Sukontapol C, Kemsen S, Chansirikarn S, Nakawiro D, Kuha O, Taemeeyapradit U. The effectiveness of a cognitive training program in people with mild cognitive impairment: A study in urban community. Asian J Psychiatr. 2018;35: 18–23.
