# Supplementary material for: Effect of matcha green tea on cognitive functions and sleep quality in older adults with cognitive decline: A randomized controlled study over 12 months
Source: PLoS One. 2024 Aug 30;19(8):e0309287. doi: 10.1371/journal.pone.0309287 (PMC11364242; doi:10.1371/journal.pone.0309287)
Supplement: S1 Table — (PDF) [file pone.0309287.s002.pdf]

S1 Table. Subsets of neurocognitive test corresponding to neurocognitive domain score used in the matcha intervention study

Neurocognitive domain score	Subsets of neurocognitive test			
Reaction time	ST			
Complex Attention	ST	SAT	CPT	
Cognitive Flexibility	ST	SAT		
Executive Function		SAT		
Social Acuity				POET
Simple Attention			CPT	

ST, stroop test; SAT, shifting attention test; CPT, continuous performance test; POET, perception of emotions test
