# Supplementary material for: Effect of matcha green tea on cognitive functions and sleep quality in older adults with cognitive decline: A randomized controlled study over 12 months
Source: PLoS One. 2024 Aug 30;19(8):e0309287. doi: 10.1371/journal.pone.0309287 (PMC11364242; doi:10.1371/journal.pone.0309287)
Supplement: S2 Table — (PDF) [file pone.0309287.s003.pdf]

S2 Table. Baseline clinical chemistry parameters of participants in the matcha and placebo groups.

Blood test	Matcha group (n = 49)	Placebo group (n = 50)
WBC, /μL	5,200 (1,600)*	5,500 (1,600)
RBC, /10^4 μL	438 (57)	434.5 (59)
Hb, g/dL	13.5 (1.1)	13.5 (1.7)
HT, %	40.7 (4.9)	40.5 (5.2)
PLT, /10^4 μL	22 (4)	21.4 (4.6)
TP, g/dL	7.3 (0.5)	7.1 (0.3)
ALB, g/dL	4.2 (0.3)	4.3 (0.2)
AST, U/L	23 (7)	20.5 (4)
ALT, U/L	18 (10)	15 (8)
ALP, U/L	79.45 (18.55)	74.9 (20.65)
LD, U/L	192 (43)	178.5 (33)
γ-GT, U/L	23 (16)	22 (15)
Total cholesterol, mg/dL	203 (45)	204 (48)
HDL, mg/dL	61.8 (21)	59.2 (25.1)
LDL, mg/dL	116 (31)	113 (39)
TG, mg/dL	130 (138)	128 (87)
BUN, mg/dL	17 (4.6)	16.15 (3.7)
Creatinine, mg/dL	0.72 (0.25)	0.69 (0.19)
Glucose, mg/dL	101 (21)	102.5 (20)
HbA1c, %	5.7 (0.6)	5.7 (0.6)
Vitamin B12, pg/mL	477.5 (229)	475.5 (247)
Folic acid, ng/mL	9.1 (3.5)	9.55 (5.15)

*median (IQR, Interquartile range)

†Mann–Whitney U test
