# Supplementary material for: Effect of matcha green tea on cognitive functions and sleep quality in older adults with cognitive decline: A randomized controlled study over 12 months
Source: PLoS One. 2024 Aug 30;19(8):e0309287. doi: 10.1371/journal.pone.0309287 (PMC11364242; doi:10.1371/journal.pone.0309287)
Supplement: S3 Table — (PDF) [file pone.0309287.s004.pdf]

Table S3. Effect of 12-month Matcha intervention on sleep quality and cogniitve functioning in per-protocol analysis

Outcome	Mixed effect model Baseline to 12 month			
	Estimate*	S.E.†	*t*-value	*P*-value
PSQI	0.89	0.54	1.62	0.108‡
ADCS-MCI-ADL	0.07	0.89	0.08	0.934
MoCA-J	0.55	0.48	1.15	0.251
MMSE-J	-0.4	0.34	-1.19	0.235
ADAS-Jcog	-0.41	0.42	-0.96	0.334
RBANS	-0.47	2.13	-0.22	0.826
CNS Vital Signs (Neurocognitive domain scores)				
Social acuity	-1.44	0.81	-1.78	0.077‡
Reaction time	20.15	18.32	1.1	0.273
Complex attention	-0.47	1.56	-0.3	0.765
Cognitive flexibility	4.31	2.99	1.44	0.151
Executive function	3.88	2.92	1.33	0.185
Simple attention	-1.24	0.78	-1.6	0.112

* Estimate: The two-group difference in mean change from baseline to 12-momth caluculated aas described in the Methods. Positive value means higher value and negative value means lower value in Matcha group comparing to placebo group, respectively.

† S.E.: Standard error

‡ $P < 0.1$
