# Supplementary material for: Effect of matcha green tea on cognitive functions and sleep quality in older adults with cognitive decline: A randomized controlled study over 12 months
Source: PLoS One. 2024 Aug 30;19(8):e0309287. doi: 10.1371/journal.pone.0309287 (PMC11364242; doi:10.1371/journal.pone.0309287)
Supplement: S4 Table — (PDF) [file pone.0309287.s005.pdf]

Table S4. Effect of 12-month Matcha intervention on neuroimaging in per-protocol analysis

Outcome	Mixed effect model Baseline to 12 month			
	Estimate*	S.E.†	*t*-value	*P*-value
Amyloid PET SUVR	0.014	0.02	0.724	0.478
MRI				
Severity of VOI atrophy	-0.036	0.03	-1.27	0.207
Exent of VOI atrophy, %	-0.350	0.64	-0.54	0.589
Ratio of VOI/GM atrophy	-0.021	0.19	-0.11	0.912
SPECT				
Severity	-0.0002	0.04	-0.01	0.996
Extent, %	0.466	1.01	0.46	0.644
Ratio	0.086	0.28	0.31	0.756

* Estimate: The two-group difference in mean change from baseline to 12-momth caluculated aas described in the Methods. Positive value means higher value and negative value means lower value in Matcha group comparing to placebo group, respectively.

† S.E.: Standard error
