# Supplementary material for: Effect of matcha green tea on cognitive functions and sleep quality in older adults with cognitive decline: A randomized controlled study over 12 months
Source: PLoS One. 2024 Aug 30;19(8):e0309287. doi: 10.1371/journal.pone.0309287 (PMC11364242; doi:10.1371/journal.pone.0309287)
Supplement: Attachment — Submitted filename: renamed_7c415.docx [file pone.0309287.s006.docx]

# COMMENTS – Manuscript PONE-D-24-02571

Title: “Effect of matcha green tea on cognitive functions and sleep quality in older adults with cognitive decline: A randomized controlled study over 12 months”

**Important note: This review pertains only to ‘statistical aspects’ of the study and so ‘clinical aspects’ [like medical importance, relevance of the study, ‘clinical significance and** **implication(s)’ of the whole study, etc.] are to be evaluated [should be assessed] separately/independently. Further please note that any ‘statistical review’ is generally done under the assumption that (such) study specific methodological [as well as execution] issues are perfectly taken care of by the investigator(s). This review is not an exception to that and so does not cover clinical aspects {however, seldom comments are made only if those issues are intimately / scientifically related & intermingle with ‘statistical aspects’ of the study}. Agreed that** **‘statistical methods’ are used as just tools here, however, they are vital part of methodology [and so should be given due importance]. I look at the manuscript in/with statistical view point, other reviewer(s) look(s) at it with different angle so that in totality the review is very comprehensive. However, there should be efforts from authors side to improve (may be by taking clues from reviewer’s comments). Therefore, please do not limit the revision only (with respect) to comments made here.**

**COMMENTS:** Kindly note that your ABSTRACT is well drafted (in my opinion), but is ‘assay type’. It is preferable [refer to item 1b of CONSORT checklist 2010: Structured summary of trial design, methods, results, and conclusions] to divide the ABSTRACT with small sections like ‘Objective(s)’, ‘Methods’, ‘Results’, ‘Conclusions’, etc. which is an accepted practice of most of the good/standard journals [including this one, though ‘The PLoS One Guidelines to Authors’ did not specify an Abstract format, it is desirable]. It will definitely be more informative then, I guess, whatever the article type may be. Since your ‘Article Type: Clinical Trial’, it very much desirable.

In addition, there are few issues/observations about which I have different opinion. Such observations/concerns are given below [if acceptable/agreed, try to incorporate them (consider positively wherever possible)]:

First is regarding choice of ‘Sample size’. One of the very important items in CONSORT guidelines is ’How sample size was determined (Item 7a)’ and expected/desired to described in ample details including all assumptions and software used. In fact, it is very surprising to note the ‘absence’ of the important term “CONSORT’’ despite that the manuscript is on ‘A randomized controlled clinical study/trial’. It is well-known that while reporting [findings from and even planning that is in ‘protocol’ of] ‘Clinical Trial’ one should follow/cover CONSORT guidelines.

In ‘Discussion’ it is just said/mentioned that “In this study, unbiased randomization was conducted, and the trial was implemented as a triple-blind study”. Regarding these important issues more discussion/detailed account is expected. Few important items {and all ‘Clinical Trials’ must follow} in CONSORT guidelines are ‘How sample size was determined (Item 7a)’,’ Random Sequence generation (Item 8a)’, ‘Allocation concealment (Item 9)’, Blinding (Item 11a). Since your article type is ‘Clinical Trial’, you are supposed to cover these items in the report or even in ‘Protocol’.

You may know that ‘Permuted Block Randomization’ ensures same group sizes (not simple randomization) and that randomization is a process [not only sequence generation] which includes ‘Allocation Concealment’}. Which ‘Randomization’ procedure is used in this study is not specified but almost equal group sizes are obtained. It must be ‘Permuted Block Randomization’ but such clear mention is desired.

In ‘Results-Participants’ section (as well as in Figure-1), you may use the word ‘screened’ in place of ‘recruited’ [As shown in **Fig 1**, we recruited 939 community-dwelling older adults aged 60–85 years and 124 participants were enrolled], in my opinion. May please confirm as word ‘recruited’ implies that 939 participated in trial. It may be known to you that when random allocation/assignment is used/done, any statistical comparison of baseline characteristics is not required. In this context [refer to Table-1], I request authors to read following note pasted from one famous standard textbook on ‘Medical Research Methodology’:

To provide a description of baseline characteristics is entirely reasonable (since it is clearly important in assessing to whom the results of the trial can be applied), however, statistical comparison of baseline characteristics when random allocation/assignment is used/done [often for good/standard/leading journals these days] is not required, because even if P-value(s) turn(s) out to be significant (while comparing baseline characteristics despite random allocation), it is, by definition, a false positive as you then are supposed to be testing ‘randomization’ then, which in any single trial may not balance all baseline characteristics (particularly when sample sizes are small). Remember that ‘randomization’ is a sort of ‘insurance’ and not a guarantee scheme. Authors may please refer to following articles:

References:

1. Stuart J. Pocock, et al., ‘Subgroup analysis, covariate adjustment and baseline comparisons in clinical trial reporting: current practice and problems’, Statistics in medicine, 2002; 21:2917–2930 [Particularly page 2927]
2. Harrington D, et al., ‘New guidelines for statistical reporting in the journal’, N Engl J Med 2019;381:285-6

[Important message (indirectly/ultimately indicated) from these articles: Never do any comparison with respect to ‘baseline’ characteristics {by applying statistical significance test(s)}, when allocation is done randomly].

However, Statistical comparison [only with respect to important/indicated variables] of baseline characteristics may be performed, to find out if analysis adjustment (say stratified analyses or else) is required with respect to these variables.

What is the purpose of Table-3? Why Between group comparison is performed separately at ‘Baseline’ and ’12-months’? What is/are interpretation of insignificant (both) P-vale(s)? Why not the comparison using ‘change scores’? In all the tables, test name is indicated only for few ‘variables/parameters’ {by giving symbol as superscript on P-value (last column)}. Is not it required to indicate test name in case of (i.e. with respect to) other/remaining variables/parameters? Or is it left for readers to guess? Remember/mind you that this is a scientific/academic document and so all details should be clearly/correctly communicated (do not take readers’ for granted). Most of the findings are reported without properly interpreting them or given relevance to study (example: page 26).

Only limitation of the study [A limitation of the current study is the small number of participants] mentioned on page 32 is agreed. However, does that mean {according to authors} there are none others? As pointed out in ‘important note’ above “This review pertains only to ‘statistical aspects’ of the study and so ‘clinical aspects’ should be assessed separately/independently. In my opinion, to make this article acceptable some amount of re-vision (re-drafting) may be needed. However, please do not limit the revision only (with respect) to comments made here. ‘Major revision’ is recommended.
