# Supplementary material for: Effect of matcha green tea on cognitive functions and sleep quality in older adults with cognitive decline: A randomized controlled study over 12 months
Source: PLoS One. 2024 Aug 30;19(8):e0309287. doi: 10.1371/journal.pone.0309287 (PMC11364242; doi:10.1371/journal.pone.0309287)
Supplement: Attachment — Submitted filename: Response to Reviewers.docx [file pone.0309287.s007.docx]

**Response to the reviewers’ comments**

**Reviewer 1:**

Thank you for your insightful suggestions, we have made several changes in the revised manuscript according to the Reviewer’s comments. These changes have enriched the context of our research, providing a clearer pathway for our readers to understand the implications of our findings.

1. Data statement – no explanation as to why data can’t be shared. States without permission from all authors but no explanation as to why all authors wouldn’t give permission. Is data owned by a third party?

**Ans:** After careful consideration and discussion among the authors, we decided to share the data in this study. The data has been uploaded to Dryad.

<https://doi.org/10.5061/dryad.2280gb61r>

We have added the following sentence.

**Page 36, lines 563-564**

The data in this study can be accessed in Dryad Digital Repository (https://doi.org/10.5061/dryad.2280gb61r).

2. Typo on page 6, line 3, double comma

**Page 5, lines 53**

**Ans:** We have corrected it in the revised manuscript.

3. Line 8 – could some context be given as to what is being classified as ‘middle age’ here.

**Ans:** According to reference (5), people aged 45-54 years were classified as “middle age.” We changed the sentences as follows in the revised manuscript.

**Page 5, lines 58-60**

Accumulating evidence suggests that lifestyle habits after middle age (approximately 45–54 years old) significantly impact the maintenance of cognitive function in older adults [3–5]

4. Within intervention section it would be useful to provide some justification as to why this dosage was decided upon.

**Ans:** As the amount of matcha in the traditional tea ceremony (Tea Otemae) is 2 g, a daily intake of matcha was set at 2 g.

We have added the following sentence.

**Page 15, lines 233-235**

As the amount of matcha in the traditional tea ceremony (Tea Otemae) is 2 g, the daily intake quantity of matcha was set at 2 g in this study.

5. For statistical analysis is there justification as to why per protocol participants were analysed rather than Intent To Treat (ITT). CONSORT recommends reporting both in parallel, so some acknowledgement of ITT would be beneficial here, were the results the same?

**Ans:** We have clarified the analytical methodologies concerning the intent-to-treat (ITT) and per-protocol (PP) in the revised manuscript. According to the ITT principle, we presented the data by ITT analysis in the original and revised manuscript. We analyzed changes in outcome variables from baseline to 12 months using a mixed-effects model using 46 participants in the matcha group and 45 in the placebo group. In addition to the ITT analysis, PP analysis was conducted, the results are shown in the S3 and S4 Tables of the revised manuscript, and corresponding modifications and textual additions have been made in the revised manuscript as follows:

**Page 21, lines 350-352**

A total of 99 participants were randomized and allocated to the matcha group (n = 49) and matched placebo group (n = 50).

**Page 22, lines 366-373**

The total number of completed questionnaires was 46 in the matcha group and 45 in the placebo group. During the intervention, each participant with MCI in the matcha and placebo groups was diagnosed with dementia at the 12-month evaluation. After excluding these two participants, the per-protocol analysis of the intervention effects included 45 and 44 participants in the matcha and placebo groups, respectively. According to the intention-to-treat principle, we analyzed the changes in outcome variables from baseline to 12 months were statistically analyzed using a mixed-effects model, with 49 and 50 participants in the matcha and placebo groups, respectively.

**Page 28, lines 422-425**

We obtained results from both intent-to-treat and per-protocol analyses. The per-protocol analysis showed that differences in social acuity and PSQI scores between the groups were -1.44 (95% CI: -2.88, 0.001; *P* = 0.077) and 0.89 (95% CI: -0.001, 1.74; *P* = 1.08), respectively (**S3** and **S4 Tables**).

6. The results are interesting and placed well within previous literature. However, there were no effects on primary outcome, and this should be acknowledged somewhere in the discussion.

**Ans:** We agree that the results of the primary outcome should be acknowledged. A corresponding statement has been added to the Discussion section. In the revised manuscript, we added the following sentences.

**Page 28, lines 419-422**

Other cognitive functions, including the primary outcomes (MoCA-J and ADCS-MCI-ADL), showed no significant changes in the matcha group compared with the placebo control group.

**Page 34, lines 524-527**

However, the matcha intervention had no effect on the primary outcomes (MoCA-J and ADCS-MCI-ADL) possibly due to their sensitivity to only slight declines in cognitive function. These tests are typically used during clinical diagnoses rather than for evaluating early-stage cognitive decline in the disease.

**Reviewer 2:**

Thank you for your crucial insights regarding the statistical elements of our study and the noted deviations from the CONSORT guidelines. These observations led us to meticulously revise the manuscript in accordance with the CONSORT standards. We believe that these adjustments have fortified the methods and results, and sharpened the conclusions, thereby enhancing the overall integrity of the manuscript.

1. Kindly note that your ABSTRACT is well drafted (in my opinion), but is ‘assay type’. It is preferable [refer to item 1b of CONSORT checklist 2010: Structured summary of trial design, methods, results, and conclusions] to divide the ABSTRACT with small sections like ‘Objective(s)’, ‘Methods’, ‘Results’, ‘Conclusions’, etc. which is an accepted practice of most of the good/standard journals [including this one, though ‘The PLoS One Guidelines to Authors’ did not specify an Abstract format, it is desirable]. It will definitely be more informative then, I guess, whatever the article type may be. Since your ‘Article Type: Clinical Trial’, it very much desirable.

**Ans:** Following the reviewer's comment, we have revised the abstract in accordance with the CONSORT guidelines

**Pages 3-4, Abstract**

**Objective**: Lifestyle habits after middle age significantly impact the maintenance of cognitive function in older adults. Nutritional intake is closely related to lifestyle habits; therefore, nutrition is a pivotal factor in the prevention of dementia in the preclinical stages. Matcha green tea powder (matcha), which contains epigallocatechin gallate, theanine, and caffeine, has beneficial effects on cognitive function and mood. We conducted a randomized, double-blind, placebo-controlled clinical study over 12 months to examine the effect of matcha on cognitive function and sleep quality.

**Methods**: Ninety-nine participants, including 64 with subjective cognitive decline and 35 with mild cognitive impairment, were randomized, with 49 receiving 2 g of matcha and 50 receiving a placebo daily. Participants were stratified based on two factors: age at baseline and *APOE* genotype. Changes in cognitive function and sleep quality were analyzed using a mixed-effects model.

**Results**: Matcha consumption led to significant improvements in social acuity score (difference: -1.39, 95% confidence interval; -2.78, 0.002) (*P* = 0.028) as evaluated by the perception of facial emotions in cognitive function. The primary outcomes, that is, Montreal Cognitive Assessment and Alzheimer’s Disease Cooperative Study Activity of Daily Living scores, showed no significant changes with matcha intervention. Meanwhile, Pittsburgh Sleep Quality Index scores indicated a trend toward improvement with a difference of 0.86 (95% confidence interval; -0.002, 1.71) (*P* = 0.088) between the groups in changes from baseline to 12 months.

**Conclusions**: The present study suggests regular consumption of matcha could improve emotional perception and sleep quality in older adults with mild cognitive decline. Given the widespread availability and cultural acceptance of matcha green tea, incorporating it into the daily routine may offer a simple yet effective strategy for cognitive enhancement and dementia prevention.

2. In addition, there are few issues/observations about which I have different opinion. Such observations/concerns are given below [if acceptable/agreed, try to incorporate them (consider positively wherever possible)]:

First is regarding choice of ‘Sample size’. One of the very important items in CONSORT guidelines is ’How sample size was determined (Item 7a)’ and expected/desired to described in ample details including all assumptions and software used.

In fact, it is very surprising to note the ‘absence’ of the important term “CONSORT’’ despite that the manuscript is on ‘A randomized controlled clinical study/trial’. It is well-known that while reporting [findings from and even planning that is in ‘protocol’ of] ‘Clinical Trial’ one should follow/cover CONSORT guidelines.

In ‘Discussion’ it is just said/mentioned that “In this study, unbiased randomization was conducted, and the trial was implemented as a triple-blind study”. Regarding these important issues more discussion/detailed account is expected. Few important items {and all ‘Clinical Trials’ must follow} in CONSORT guidelines are ‘How sample size was determined (Item 7a)’,’ Random Sequence generation (Item 8a)’, ‘Allocation concealment (Item 9)’, Blinding (Item 11a). Since your article type is ‘Clinical Trial’, you are supposed to cover these items in the report or even in ‘Protocol’.

You may know that ‘Permuted Block Randomization’ ensures same group sizes (not simple randomization) and that randomization is a process [not only sequence generation] which includes ‘Allocation Concealment’}. Which ‘Randomization’ procedure is used in this study is not specified but almost equal group sizes are obtained. It must be ‘Permuted Block Randomization’ but such clear mention is desired.

**Ans:** According to the reviewer’s comment, we added the following sentences and references in the Materials and Methods: “Study design,” “Randomization,” and “Participants” of the revised manuscript.

**Page 8, line 110－page 9, line 123**

**Study design**

A 12-month intervention was executed employing a randomized, double-blind, placebo-controlled, parallel-group design. Before the initiation of the study, the clinical trial was registered with the UMIN Clinical Trials Registry (UMIN-CTR), which is recognized by the International Committee of Medical Journal Editors (ICMJE) as an approved registry. This study was conducted as a randomized controlled trial, adhering strictly to the CONSORT guidelines. The required sample size was calculated using G*Power 3 software (Heinrich Heine University, Düsseldorf, Germany). We chose to assess the difference between the medians of two independent groups using the Wilcoxon or Mann–Whitney test. With the parameters set as two-tailed, logistic distribution, an effect size of 0.7, a significance level of 0.05, and a power of 0.8, it was determined that each group required 31 cases. Anticipating a dropout rate of 7 of 40 cases per group, we established a target sample size of at least 33 cases per group to validate the robustness of our findings (details are described in **S1 File**).

**Page 9, lines 124-133**

**Randomization**

**Sequence generation**

Eligible participants with SCD or MCI were randomized using a computer-generated sequence. Participants were stratified based on two stratification factors, age at baseline (≥ 74 years / < 74 years) and *APOE* genotype (ε4 positive/negative). Participants were stratified based on age at 74 years because a 10-year AD risk is higher in people ≥ 74 years old than in those < 74 years old [30]. Allocation to either the control or intervention group was intended to be randomized in a 1:1 ratio using computerized minimization, which aims to minimize differences between groups with respect to the stratification factors [31].

**Page 9, line 134-page 11, line 166**

**Allocation concealment mechanism**

Participants underwent randomization using a dynamic program implemented via a Microsoft Excel macro, employing simple randomization rather than permuted block randomization. Upon running the program, the participants were assigned to either Group A or Group B. However, the program did not reveal which was the intervention or control group. The randomization table created by the program was retained until all baseline stratification factors were fully aligned and participant inclusion was complete, ensuring concealment of the allocation.

All eligible participants who provided informed consent and met the predefined inclusion criteria underwent randomization, a process administered at the discretion of the study site personnel overseeing recruitment and medical interviews. Subsequently, the study site transmitted a stratification factor response form to an independent allocation manager who was responsible for the allocation process, ensuring separation from the outcome evaluation analysis. The allocation manager utilized a dynamic randomization program incorporating provided stratification factors to assign participants to their respective groups based on the program's output. Physicians and nurses responsible for participant inclusion and symptom assessment remained blinded to group assignments.

The allocation sequence utilized a stratified approach, considering study site and medication compliance, employing a dynamic allocation method with the minimization technique. Concealment of randomization details from data management personnel and statistical analysts was upheld throughout the study while ensuring the database remained publicly accessible. The randomization list was securely maintained by designated allocation personnel throughout the study, mitigating any potential influence from principal investigators involved in the research.

**Blinding**

Throughout the study duration, participants were unaware of whether the capsules they consumed contained matcha or placebo until the study's conclusion was obtained. Physicians, nurses, and principal investigators at the study site were informed that the participants had been assigned to provisional groups (Groups A and B), yet remained uninformed about the specific allocation to matcha or placebo groups until the randomization list was released after the study period. No member of the research team knew the allocated sequence until the end of the statistical analysis after the database was locked.

**Page 11, lines 167-170**

**Participants**

Community-dwelling older adults aged 60–85 years were recruited and assessment for eligibility was performed at the University of Tsukuba Hospital (Tsukuba, Ibaraki, Japan) and Memory Clinic (Toride, Ibaraki, Japan) (**Fig 1**).

3. In ‘Results-Participants’ section (as well as in Figure-1), you may use the word ‘screened’ in place of ‘recruited’ [As shown in **Fig 1**, we recruited 939 community-dwelling older adults aged 60–85 years and 124 participants were enrolled], in my opinion. May please confirm as word ‘recruited’ implies that 939 participated in trial. It may be known to you that when random allocation/assignment is used/done, any statistical comparison of baseline characteristics is not required. In this context [refer to Table-1], I request authors to read following note pasted from one famous standard textbook on ‘Medical Research Methodology’.

**Ans:** I would like to thank you very much for your valuable comments and recommendation of the textbook. I think that this is the book by Joseph Abramson.

I am sorry for the misuse of the term. We have corrected the word “screened” to “recruited.” We have also revised **Fig 1** and **Table 1**.

4. To provide a description of baseline characteristics is entirely reasonable (since it is clearly important in assessing to whom the results of the trial can be applied), however, statistical comparison of baseline characteristics when random allocation/assignment is used/done [often for good/standard/leading journals these days] is not required, because even if P-value(s) turn(s) out to be significant (while comparing baseline characteristics despite random allocation), it is, by definition, a false positive as you then are supposed to be testing ‘randomization’ then, which in any single trial may not balance all baseline characteristics (particularly when sample sizes are small). Remember that ‘randomization’ is a sort of ‘insurance’ and not a guarantee scheme. Authors may please refer to following articles:

References:

1.	Stuart J. Pocock, et al., ‘Subgroup analysis, covariate adjustment and baseline comparisons in clinical trial reporting: current practice and problems’, Statistics in medicine, 2002; 21:2917–2930 [Particularly page 29, 27]

2.	Harrington D, et al., ‘New guidelines for statistical reporting in the journal’, N Engl J Med 2019;381:285-6

[Important message (indirectly/ultimately indicated) from these articles: Never do any comparison with respect to ‘baseline’ characteristics {by applying statistical significance test(s)}, when allocation is done randomly]. However, Statistical comparison [only with respect to important/indicated variables] of baseline characteristics may be performed, to find out if analysis adjustment (say stratified analyses or else) is required with respect to these variables.

**Ans:** Thank you for your valuable comments on the description of baseline characteristics. We agree that statistical comparison of baseline characteristics is not required as random allocation/assignment is used/done in this study. According to the Reviewer’s comment, we revised **Table** **1** and **Table** **2** in which all *P*-value and statistical test names were deleted, and the *P*-value in two stratification factors, age and *APOE* genotype are indicated in the text as follows:

**Page 25, line 387-page 26, line 388**

There was no difference in the average age (*P* = 0.907) and percentage of *APOE*4 carriers (*P* = 0.876) between the groups, indicating random assignment.

5. What is the purpose of Table-3? Why Between group comparison is performed separately at ‘Baseline’ and ’12-months’? What is/are interpretation of insignificant (both) P-vale(s)? Why not the comparison using ‘change scores’? In all the tables, test name is indicated only for few ‘variables/parameters’ {by giving symbol as superscript on P-value (last column)}. Is not it required to indicate test name in case of (i.e. with respect to) other/remaining variables/parameters? Or is it left for readers to guess? Remember/mind you that this is a scientific/academic document and so all details should be clearly/correctly communicated (do not take readers’ for granted). Most of the findings are reported without properly interpreting them or given relevance to study (example: page 26).

**Ans: Table 3** indicates the baseline and 12-month characteristics of neuroimaging amyloid PET. We agree that a group comparison at “Baseline” and “12-months” is not required for the purpose of this study, and Table 5, in which changes between baseline and 12 months are indicated, is enough to indicate the effect of the matcha intervention in this study.

Amyloid load is important information in the diagnosis of Alzheimer’s disease. The cut-off value of SUVR in Alzheimer’s disease vs. non-cognitive control is usually 1.11. **Table 3** may be informative for readers to know the pathophysiological status of participants in terms of amyloid pathology in Alzheimer’s disease.

According to the reviewer’s comment, we have removed *P*-values and the names of statistical tests from **Table 3**. Additionally, we revised the Results section of the manuscript to enhance the interpretation and relevance of the data.

6. Only limitation of the study [A limitation of the current study is the small number of participants] mentioned on page 32 is agreed. However, does that mean {according to authors} there are none others? As pointed out in ‘important note’ above “This review pertains only to ‘statistical aspects’ of the study and so ‘clinical aspects’ should be assessed separately/independently. In my opinion, to make this article acceptable some amount of re-vision (re-drafting) may be needed. However, please do not limit the revision only (with respect) to comments made here. ‘Major revision’ is recommended.

**Ans:** According to several comments of the reviewers, including two other reviewers, a major revision has been made. We rewrote the manuscript and added the tables. We are truly thankful for the opportunity to refine our manuscript based on the thoughtful feedback provided. The following sentences were added to the Discussion section.

**“**A limitation of the current study” in the revised manuscript.

**Page 35, lines 540-544**

The observed PSQI score improvement within the intervention group suggests a potential influence of matcha consumption on sleep quality. However, future studies should employ more objective measures beyond the PSQI to comprehensively assess sleep architecture, including quantification of REM and non-REM sleep stages.

**Reviewer 3:**

Thank you for your thorough review and valuable insights into our manuscript. During the revision process, we have diligently addressed each point raised by the reviewers to ensure that all concerns are comprehensively resolved.

1. Introduction is well written with relevant links to supporting green tea literature. It would be useful to include rationale for the intervention timeframe (12 months). There does seem to be some literature on matcha tea interventions that haven't been included, which is more relevant than the included green tea literature - for example a paper by Sakurai (2020) used matcha for a 12 week-intervention in elderly Japanese individuals.

**Ans:** According to the reviewer’s comment, we have added the following sentences and references in the revised manuscript.

**Page 6, lines 77-81**

Randomized, double-blind, placebo-controlled trials have reported beneficial effects of matcha tea on cognitive function [6,7,13]. However, the intervention periods in these studies were relatively short, lasting only 12 weeks. Longer intervention periods, extending several months or more, are required to comprehensively evaluate the effects of matcha consumption as a lifestyle habit.

6. Baba Y, Inagaki S, Nakagawa S, Kobayashi M, Kaneko T, Takihara T. Effects of Daily Matcha and Caffeine Intake on Mild Acute Psychological Stress-Related Cognitive Function in Middle-Aged and Older Adults: A Randomized Placebo-Controlled Study. Nutrients. 2021;13.

7. Baba Y, Kaneko T, Takihara T. Matcha consumption maintains attentional function following a mild acute psychological stress without affecting a feeling of fatigue: A randomized placebo-controlled study in young adults. Nutr Res. 2021;88: 44–52.

13. Sakurai K, Shen C, Ezaki Y, Inamura N, Fukushima Y, Masuoka N, et al. Effects of matcha green tea powder on cognitive functions of community-dwelling elderly individuals. Nutrients. 2020;12. doi:10.3390/nu12123639

2. Page 7 line 5 - missing reference for frequency of green tea consumption. It would also be useful here to have a brief explanation of this finding and the relation to this trial.

**Ans:** We rewrote the following sentences and references in the revised manuscript.

**Page 5, line 67-Page 6, line 76**

A cross-sectional observational study revealed that the risk of cognitive decline, indicated by a score of < 26 on the Mini Mental State Examination-Japanese version (MMSE-J), was significantly lower in the group that consumed 200 mL or more of green tea per day, indicating a positive correlation between regular green tea consumption and maintained cognitive function [11]. With a cut-off point of 26, the odds ratios for the different frequencies of green tea consumption were 0.42 for 2–3 cups per day, compared with 1.00 (reference) for 3 cups per week. In a traditional tea ceremony (Tea Otemae), 2 g of matcha contains 170 mg of catechins. In comparison, 2–3 cups of green tea contain 135–200 mg of catechins, with green tea providing 67.5 mg of catechins per 100 mL [11,12].

11. Kuriyama S, et al. Green tea consumption and cognitive function: a cross-sectional study from the Tsurugaya Project. Am J Clin Nutr. 2006;83: 355–361.

12. Khokhar, S.; Magnusdottir, S.G.M. Total Phenol, Catechin, and Caffeine Contents of Teas Commonly Consumed in the United Kingdom. J. Agric. Food Chem. 2002, 50, 565–570.

3. Page 7 line 8 - missing reference for sleep disturbance/disorder stats, it would be more useful here to have the stats for sleep disorder prevalence in Japan rather than the US.

**Ans:** We rewrote the sentences and references in the revised manuscript as follows:

**Page 6, lines 82-84**

Approximately 20% of adults in Japan experience sleep disturbance [14,15], and 50–70 million people have sleep disorders in the United States [16].

14. Doi, Y.; et al. Prevalence of Sleep Disturbance and Hypnotic Medication Use in Relation to Sociodemographic Factors in the General Japanese Adult Population. J. Epidemiol. 2000, 10, 79–86.

15. Kim, K.et al; Ogihara, R. An Epidemiological Study of Insomnia among the Japanese General Population. Sleep 2000, 23, 41–47.

16. Committee on Sleep Medicine and Research. Sleep Disorders and Sleep Deprivation: An Unmet Public Health Problem; Colten, H.R., Altevogt, B.M., Eds.; National Academies Press: Washington DC, 2006; ISBN 9780309101110.

4. I am interested to know why age was stratified as >74 years old/ ≥74 years old, when the age range was 60-85 years. Is there a reason that 74 was chosen rather than 72? A brief comment at Page 9 line 2 would be useful.

**Ans:** According to the reviewer’s comment, we added the following sentences and a reference in the Materials and Methods of the revised manuscript.

**Page 9, lines 127-130.**

Participants were stratified based on an age cut-off of 74 years because the 10-year AD risk is higher in people ≥ 74 years old than in those < 74 years old [30].

30. Livingston, G.; et al. Dementia Prevention, Intervention, and Care: 2020 Report of the Lancet Commission. Lancet 2020, 396, 413–446.

6. Page 9 Line 16 - points 3 and 4 of the eligibility criteria are the same, please check.

**Ans:**

Due to duplicate sentences, point 4, (4) in the original manuscript, was deleted from the revised manuscript. (**Page 11, line175-176 in the revised manuscript）**

7. Within eligibility criteria, you mention that participants could not consume more that 7 cups of matcha per week, there is a comment within the discussion that the study did not impose restrictions on green tea intake during the trial, why was this decided? Did you collect information on habitual intake of green tea/matcha during the trial? If so, were there any differences in consumption between treatment groups? High consumption in the placebo group could explain lack of findings.

**Ans:** Drinking seven cups of matcha a week is equivalent to consuming 2 g of matcha every day, which is the same amount of matcha used in this intervention study. Hence, participants drinking seven cups of matcha were excluded.

When we recruited the participants, information on habitual intake of green tea and matcha was collected, however, we did not compare the intervention effects in participants by habitual intake of green tea. In this study, we used one of the highest quality matcha, “Hojin no shiro” (ITO EN, LTD). Its cultivation method differs from that of conventional green tea, as the tea leaves designated for matcha production are protected from sunlight prior to harvesting. As amount of tea constituents, especially theanine and catechins, are higher than those in green tea we drink in everyday life in Japan. Additive effects of matcha consumption could be expected in this study.

8. Intervention information - I'd be interested to know what 2 g of matcha equates to in comparison with consuming as a tea and/or how the catechin/theanine/caffeine content compares with green tea. I think this is particularly interesting given the citation of various green tea literature and the decision to exclude participants consuming 7+ cups matcha each week.

**Ans:** As previously mentioned, 2 g of matcha contains 170 mg of catechins, while green tea contains 67.5 mg of catechins per 100 mL [Khokhar, S et al]. In terms of catechin content, a single cup of matcha is equivalent to 250 mL (2-3 cups) of green tea. It should be noted that various types of green tea exist, and not all are directly comparable to matcha. The most expensive green tea, known as “Gyokuro,” is rarely consumed in daily life due to its high cost, whereas more common varieties of green tea are frequently consumed.

According to the reviewer’s comment, we added the following sentences in the revised manuscript.

**Page 35, lines 532-537**

Participants drinking seven cups of matcha were excluded since it is equivalent to consuming 2 g of matcha every day, which is the same amount of matcha as in this intervention study. In terms of catechin content, a single cup of matcha is equivalent to 250 mL (2–3 cups) of green tea; however, it should be noted that various types of green tea exist, and not all are directly comparable to matcha.

9. Page 16 Line 2 - Please provide comment on if the repeated freezing/thawing of samples may have impacted the immunoassay results. Was this per protocol? It would also be useful here to include details of the blood sampling process. It would also be useful to include some information about the biomarkers assessed (e.g. assessing vitamin status).

**Ans:** According to the reviewer’s comment, we added the following sentences in the revised manuscript.

**Page 18, lines 292-293**

Freezing and thawing up to three times did not affect the results of the immunoassay.

10. Page 23 (Authors: I think this is Page 13) Line 7 - compliance - earlier a pill count was mentioned, do you have the information on treatment compliance based on this? Was there a compliance cut off used for including participants in analysis?

**Ans:** Intake compliance was assessed by counting the number of capsules that remained in the bag during the 3-, 6-, 9-, and 12-month visits. A compliance cut-off was not set in this study. The intake rate was calculated from the number of capsules left in the bag. Both at the University of Tsukuba and at Memory Clinic Toride, the intake rates were 98-99%. In addition to counting of capsules, we evaluated intake compliance by measuring theanine concentration in platelets [Scheid, L.; et al.], and there was a significant increase in platelet theanine levels in the matcha group compared to the placebo group.

Scheid, L.; et al. Kinetics of L-theanine uptake and metabolism in healthy participants are comparable after ingestion of L-theanine via capsules and green tea. J. Nutr. 2012, 142, 2091–2096.

According to the reviewer’s comment, we added the following sentences in the results of the revised manuscript.

**Page 26, lines 399-401**

Intake compliance was assessed by counting the number of capsules that remained in the bag at the 3-, 6-, 9-, and 12-month visits. The intake rates were 98–99% throughout the intervention periods.

11. Within sample size calculation information, I don't think it is clear why a medium-large effect size (cohen's d = 0.7) was used. Was this based off previous green tea literature? Please expand.

**Ans:** As detailed in the **S1 file** (Supporting Materials & Methods), the sample size was determined as described in section 2.1 ("Statistical Evaluation for the Number of Cases") on page 3. The meta-analysis results from 3 studies (page 7: also indicated below) indicated that with a standard deviation (SD) of 3.5 for the MoCA score, the minimum difference between groups was 2.5, leading to an effect size (d) of 0.71. For an SD of 4.0, the difference increased to 3.0, resulting in an effect size of 0.75. Given that 33 cases per group demonstrated significant results, the initial effect size assumption of 0.7 used to set the sample size was confirmed to be appropriate.

Tsolaki M, Kounti F, Agogiatou C, Poptsi E, Bakoglidou E, Zafeiropoulou M, et al. Effectiveness of nonpharmacological approaches in patients with mild cognitive impairment. Neurodegener Dis. 2011;8: 138–145.

Marzolini S, Oh P, McIlroy W, Brooks D. The effects of an aerobic and resistance exercise training program on cognition following stroke. Neurorehabil Neural Repair. 2013;27: 392–402.

Sukontapol C, Kemsen S, Chansirikarn S, Nakawiro D, Kuha O, Taemeeyapradit U. The effectiveness of a cognitive training program in people with mild cognitive impairment: A study in urban community. Asian J Psychiatr. 2018;35: 18–23.

12. Whilst this is recognized several times, consider highlighting each time that the PSQI finding is a trend only to be careful when over interpreting this finding.

**Ans:** We agree with the reviewer’s comment, and the following sentences were added to the Discussion section.

“A limitation of the current study” in the revised manuscript.

**Page 35, lines 540-544**

The observed PSQI score improvement within the intervention group suggests a potential influence of matcha consumption on sleep quality. However, future studies should employ more objective measures beyond the PSQI to comprehensively assess sleep architecture, including quantification of REM and non-REM sleep stages.

13. I also found a few minor typos:

Page 6 Line 3 – extra comma

Page 20 line 4 – matcha misspelled

**Ans:** We have corrected them in the revised manuscript.

**Page 5, lines 53** – extra comma: corrected

**Page 22, line 368** – matcha misspelled: corrected
