# Supplementary material for: Effect of matcha green tea on cognitive functions and sleep quality in older adults with cognitive decline: A randomized controlled study over 12 months
Source: PLoS One. 2024 Aug 30;19(8):e0309287. doi: 10.1371/journal.pone.0309287 (PMC11364242; doi:10.1371/journal.pone.0309287)
Supplement: Attachment — Submitted filename: renamed_f977b.docx [file pone.0309287.s008.docx]

**Answer to the reviewer’s comments**

**Reviewer 2:**

**COMMENTS:**

Since all of the comments made on earlier draft are considered positively & mostly attended satisfactorily, I recommend the acceptance after minor revision. Although many modifications could be seen clearly, few details/modifications given while addressing comments were/are not very convincing [example: account regarding choice of ‘Sample size’].

‘Minor revision’ is recommended so that authors can re-visit earlier comments and further modify by taking clues from earlier comments. However, please do not limit the revision only (with respect) to comments made there. More improvement is expected {note that ‘clinical implications’ are valuable}.

**ANSWER:**

We are sincerely grateful for the opportunity to further improve our manuscript. In response to your valuable feedback, we have deepened the discussion regarding the clinical implications and limitations of our findings. We have incorporated a paragraph that explicitly outlines how our results concerning the effects of matcha consumption may contribute to dementia prevention strategies (page 35, line 554 – page 36, line 561). This paragraph also discusses the implications of our findings for the field of preventive medicine, thereby strengthening the connection between our research and its practical applications.

Regarding the chosen sample size, we acknowledge your feedback indicating that our rationale was not sufficiently articulated. To address this, we have anchored our determination of sample size in a robust statistical rationale, incorporating considerations such as power analysis and effect size, consistent with the guidelines recommended for studies of this nature. We have elaborated on this discussion in the revised manuscript, providing a more detailed justification and citing studies [30–32] employing analogous methodologies (page 8, line 120 – page 9, line 126).

We have diligently reviewed the manuscript to ensure consistency of content. Additionally, we have verified the references to ensure they are complete and correct, deleted duplicated references, and added three new references.

We added the following sentences and references in the Materials and Methods:

**Page 8, line 120** – **Page 9, line 126**

A meta-analysis was conducted to ascertain whether the effect size and statistical power were commensurate with the determined sample size, referencing three intervention studies that employed the Montreal Cognitive Assessment (MoCA) as the evaluative criterion [29–31]. Although the efficacy indices in this study vary slightly from those in previous research, we concluded that observing the anticipated effects outlined in the meta-analysis would render the data from all 66 patients a significant evidence base for preparing the subsequent validation study.

**References**

30.	Tsolaki M, Kounti F, Agogiatou C, Poptsi E, Bakoglidou E, Zafeiropoulou M, et al. Effectiveness of nonpharmacological approaches in patients with mild cognitive impairment. Neurodegener Dis. 2011;8: 138–145.

31.	Marzolini S, Oh P, McIlroy W, Brooks D. The effects of an aerobic and resistance exercise training program on cognition following stroke. Neurorehabil Neural Repair. 2013;27: 392–402.

32.	Sukontapol C, Kemsen S, Chansirikarn S, Nakawiro D, Kuha O, Taemeeyapradit U. The effectiveness of a cognitive training program in people with mild cognitive impairment: A study in urban community. Asian J Psychiatr. 2018;35: 18–23.

**Page 9, lines 132** – **134**

Anticipating a dropout rate of 7 of 40 recruited cases per group, we established a target sample size of at least 33 cases per group to validate the robustness of our findings (details are described in **S1 File**).

We added the following sentences in Discussion.

**Page 35, line 554** – **Page 36, line 561**

The beneficial effects of matcha consumption revealed in this study may contribute to dementia prevention strategies, as these are relatively easy lifestyle changes to implement in daily life. Matcha consumption improved facial emotion recognition, as assessed by a computerized neurocognitive battery, since conventional psychological tests may not be suitable for evaluating cognitive decline in very mild cases. Thus, more sensitive neuropsychological testing, along with intervention strategies that are easy for elderly people to maintain, is necessary to assess the efficacy of these interventions in dementia prevention.
